# Risk of diabetes and hypertension in a population with alcohol use disorders

**DOI:** 10.1186/s12889-024-18318-y

**Published:** 2024-03-21

**Authors:** Pei-Ying Tseng, Fung-Chang Sung, Chih-Hsin Muo, Yu-Ching Lan, Yih-Ing Hser, Sarina Hui-Lin Chien, Jong-Yi Wang

**Affiliations:** 1 Department of Public Health, China Medical University, 406 Taichung, Taiwan; 2 Department of Medicine, Lee’s General Hospital, 358 Yuanli, Taiwan; 3 Management Office for Health Data, China Medical University Hospital, 404 Taichung, Taiwan; 4 Department of Health Services Administration, China Medical University College of Public Health, 406 Taichung, Taiwan; 5 Department of Food Nutrition and Health Biotechnology, Asia University, 413 Taichung, Taiwan; 6 Expert Labs, IBM Taiwan Corporation, 110 Taipei, Taiwan; 7 Department of Psychiatry and Biobehavioral Sciences, University of California Los Angeles, 90095 Los Angeles, CA USA; 8 Graduate Institute of Biomedical Sciences, China Medical University, Taichung, Taiwan; 9 Department of Health Services Administration, China Medical University, 100 Jingmao Rd. Sec. 1, Beitun Dist, 406 Taichung, Taiwan

**Keywords:** Alcohol, Hypertension, Diabetes, Retrospective cohort study, Chronic disorders

## Abstract

**Background:**

A population-based follow-up study assessing the risk of developing hypertension and diabetes associated with alcohol use disorder (AUD) is crucial. We investigated this relationship by using insurance claims data from Taiwan.

**Methods:**

From the claims data, an AUD cohort (*N* = 60,590) diagnosed between 2000 and 2006 and a non-AUD comparison cohort (*N* = 60,590) without the diagnosis of hypertension or diabetes at baseline were established and matched by propensity scores estimated by baseline demographic status and the Charlson comorbidity index (CCI). We assessed the incidence rates of hypertension and/or diabetes at the end of 2016 and used Cox’s method to estimate the related hazard ratios (HRs) and 95% confidence intervals (CIs).

**Results:**

Relative to the comparison cohort, the AUD cohort had an approximately 1.70-fold higher incidence of hypertension (35.1 vs. 20.7 per 1,000 person-years), with an adjusted HR (aHR) of 1.72 (95% CI: 1.68–1.76), 2.16-fold higher incidence of diabetes (20.2 vs. 9.36 per 1,000 person-years), with an aHR of 2.18 (95% CI: 2.11–2.24), and 1.91-fold higher incidence of both diabetes and hypertension (10.3 vs. 5.38 per 1,000 person-years) with an aHR of 2.02 (95% CI: 1.94–2.10). The incidence rates of all outcomes were greater in men than in women, whereas the HRs were greater for AUD in women than for AUD in men relative to the respective comparison patients. The risk increased further for subjects with CCI ≥ 1, which was higher in the AUD cohort.

**Conclusions:**

The increased risk of developing diabetes and hypertension in patients with AUD, especially the differences noted according to gender, indicates that clinicians should address potential comorbidities in these patients.

## Background

Both diabetes and hypertension are chronic diseases prevalent worldwide [1]. The World Health Organization recently predicted that the global population with diabetes will reach 642 million by 2040, resulting in massive medical and economic burdens [2]. Diabetes is an important disease that is associated with the possible development of chronic kidney disease, end-stage renal disease, heart attack, and stroke [3, 4]. Approximately 1.2–1.4 billion people worldwide have been diagnosed with hypertension, which is an important risk factor for heart attack, stroke, kidney disorders, and death [5].

The development of diabetes, hypertension, or other disorders has been associated with alcohol consumption in many studies [6–20]. Alcohol is a popular psychoactive substance, and its recreational use plays an important social role in the daily lives of people in most cultures. Studies have found that moderate alcohol consumption has health benefits but also risks [6, 7, 9, 16, 21]. One or two standard-sized drinks per day are considered moderate use, whereas three or more drinks per day are considered heavy use [21, 22]. Studies on the health impacts of heavy alcohol use emphasize severe consequences, such as liver damage, heart disease, mental health problems, nerve damage, and even cancer.

AUD is a chronic condition that is characterized by an inability to control or stop alcohol use despite its negative consequences. AUD is diagnosed using the DSM-V criteria and involves symptoms such as excessive drinking, unsuccessful attempts to cut down alcohol consumption, and continued use of alcohol despite its adverse effects. The severity of this condition ranges from mild to severe based on the number of criteria met. The diagnosis of AUD in a clinical setting involves a comprehensive assessment considering individual health, social factors, and potential co-occurring conditions.

Previous studies examining the risks of diabetes or hypertension associated with alcohol use have been limited to case-control analyses, cross-sectional observational studies, or blood pressure measurements in alcohol consumption surveys or trials [6–20]. In a systematic review study, Knott et al. analyzed the data of 125,926 type 2 diabetes patients from 38 observational studies and concluded that moderate alcohol use could reduce the risk of diabetes in a dose–response relationship [7]. Moderate alcohol intake may reduce blood pressure, whereas higher levels of intake elevate blood pressure in both men and women. In a survey in the USA, Esser and colleagues found that 10.2% of excessive drinkers, 10.5% of binge drinkers, and 1.3% of non-binge drinkers were alcohol dependent [22]. Individuals who drink heavily can become dependent on alcohol or develop other types of disorders. Individuals with alcohol use disorder (AUD) are at increased risk of subsequent health impairments. However, no population study has evaluated the risks of concurrently developing both hypertension and diabetes, specifically for individuals with AUD [23].

Long-term follow-up periods are required to assess patients with AUD until diabetes and/or hypertension are diagnosed. Performing a prospective cohort study to confirm the development of diabetes or hypertension in alcohol users is expensive and complex. However, it is important to evaluate the risk of developing diabetes and hypertension in individuals with AUD to prevent subsequent development of these and other health conditions. Therefore, in this study, we used population-based insurance claims data obtained from the National Health Insurance Program of Taiwan to establish cohorts with and without AUD to assess the risks of developing diabetes and hypertension.

## Methods

### Data source

This study used claims data from the Longitudinal Health Insurance Database (LHID) of Taiwan obtained from the National Health Research Institutes with authorization from the Ministry of Health and Welfare. The LHID documented the medical treatment records of beneficiaries, including information on outpatient visits, inpatient care, prescriptions of treatments and medications, and costs of care, from 1996 to 2016. Diseases in the claims data were coded using the International Classification of Diseases, Ninth Revision, Clinical Modification (ICD-9-CM) from 1996 to 2015 and the 10th revision (ICD-10-CM) in 2016. To protect personal information, personal identifications in the LHID were recoded into surrogate numbers before the database was released to researchers. This study received IRB approval by the Research Ethics Committee of the China Medical University and Hospital, Taiwan (CMUH-104-REC2-115). Requirement for informed consent was waived by the Ethics Committee of the China Medical University and Hospital, Taiwan, because patient data were collected without patient identification, to protect patient privacy.

### Study cohorts

From the claims data, we identified patients with AUD (ICD-9-CM: 291, 303.0, 303.9, 305.0, and A215; ICD-10-CM: F10.3-F10.9, F10.0, F10.1, and F10.2) who were newly diagnosed between 2000 and 2006 for the AUD cohort. Cases diagnosed between 1996 and 1999 were excluded from the study. The date of the AUD diagnosis was defined as the index date. Patients with AUD aged ≤ 20 years and those with a history of hypertension (ICD-9-CM: 401–405) and/or diabetes (ICD-9-CM: 250) at baseline were excluded. The comparison cohort was determined using the following procedure. For each AUD case, two individuals were randomly selected from the claims data without a diagnosis of AUD during the same period and matched by propensity scores. Using logistic regression, propensity scores were assessed according to age (20–39, 40–64, and 65 + years), gender, Charlson comorbidity index (CCI) score (0 and > 1), urbanization level of residence area (1, 2, 3, and 4–7), monthly income level (<$15,000 NTD, $15,001–$20,000 NTD, and >$20,001 NTD), and occupation (white collar, blue collar, and other). Urbanization level “1” correlates to the most urbanized areas, and level “7” refers to highly rural areas.

CCI scores were calculated by weighting 1 for myocardial infarction, congestive heart failure, peripheral vascular disease, cerebrovascular disease, dementia, chronic pulmonary disease, connective tissue disease, ulcerous disease, and mild liver disease; 2 for hemiplegia, moderate or severe renal disease, any tumor, leukemia, and lymphoma; 3 for moderate or severe liver disease; and 6 for metastatic solid tumors and AIDS.

All study subjects were followed up from the index date until the development of hypertension, diabetes, or both. Those who did not develop hypertension or diabetes were followed until the date of death, withdrawal from the LHID, or the end of 2016. The number of person-years of follow-up was estimated for each participant.

### Statistical analysis

First, we compared the distribution of the baseline characteristics between the two cohorts and performed a statistical analysis of the standardization differences, including age, gender, urbanization level, monthly income, occupation, and CCI score. The Kaplan–Meier method was used to calculate and plot the cumulative incidence of hypertension, diabetes, and both, and the differences were examined using a log-rank test. Sex-specific incidence rates of hypertension, diabetes, or both were estimated in both cohorts. Cox proportional hazards regression analysis was used to estimate the hazard ratio (HR) and 95% confidence interval (CI) for hypertension, diabetes, or both, by gender. A multivariate Cox model was used to measure the adjusted hazard ratio (aHR) after controlling for age, gender, urbanization, monthly income, occupation, and CCI score. We further estimated the risk of developing hypertension, diabetes, or both in both cohorts using CCI (0 and ≥ 1) to evaluate the joint effect of AUD and CCI score levels. All statistical analyses were performed using the SAS statistical software (version 9.4 for Windows; SAS Institute, Inc., Cary, N.C., USA).

## Results

At baseline, the AUD cohort (*N* = 60,590) and the comparison cohort (*N* = 60,590) were similar in terms of age, gender, urbanization level, monthly income, occupation, and CCI score (no standardization difference > 0.5) (Table 1). Nearly 85% of the study population was male, and more than half of the population was under 40 years of age. The study population was more likely to live in urbanized areas and be white collar workers with lower incomes, but < 20% of both cohorts had a CCI of ≥ 1.

**Table 1:** Demographic characteristics and Charlson Comorbidity Index (CCI) compared between alcohol use disorder (AUD) cohort and comparison cohort

	AUD		Comparison		Standardization difference
	*N* = 60,590		*N* = 60,590		
	n	%	n	%	
Age, years					
20–39	32,149	53.1	32,114	53.0	0.001
40–64	26,708	44.1	27,065	44.7	0.012
65+	1733	2.86	1411	2.33	0.033
Mean (SD)	39.8	10.9	39.8	11.5	0.005
Gender					
Men	51,175	84.5	51,087	84.3	0.004
Women	9415	15.5	9503	15.7	0.004
Urbanization					
1 (highest)	12,438	20.5	12,200	20.1	0.010
2	18,115	29.9	183.21	30.2	0.007
3	11,165	18.4	10,953	18.1	0.009
4–7 (lowest)	18,872	31.2	19,116	31.6	0.009
Monthly income, NTD					
$0–$10,000	21,406	35.3	21,320	35.2	0.003
$10,001–$20,000	26,767	44.2	27,413	45.2	0.021
>$20,001	12,417	20.5	11,857	19.6	0.023
Occupation					
White collar	16,994	28.1	16,740	27.6	0.009
Blue collar	22,224	36.7	22,597	37.3	0.013
Others	21,372	35.3	21,253	35.1	0.004
CCI score					
0	49,856	82.6	50,256	82.9	0.017
≥ 1	10,734	17.4	10,334	17.1	0.017

**Fig. 1:**
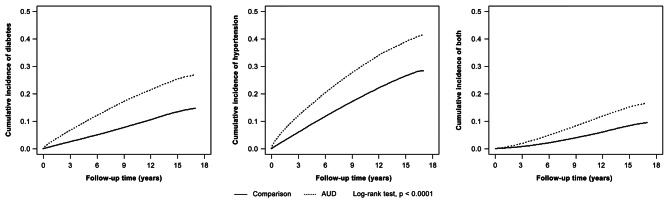
Kaplan–Meier method estimated cumulative incidences of hypertension, diabetes, and both hypertension and diabetes compared between the alcohol use disorders and comparison cohorts

By the end of the follow-up period, the cumulative incidence rates of diabetes, hypertension, and both diabetes and hypertension were 12.19%, 12.97%, and 7.10%, respectively, which were higher in the AUD cohort than in the comparison cohort (Fig. 1).

Table 2 shows that, relative to the comparison cohort, the AUD cohort had a 2.15-fold higher incidence of diabetes (20.18 vs. 9.36 per 1,000 person-years), with an aHR of 2.18 (95% CI: 2.11–2.24). Hypertension incidence was 1.7-fold higher in the AUD cohort than in the comparison cohort (35.11 vs. 20.65 per 1,000 person-years), with an aHR of 1.72 (95% CI: 1.68–1.76). The incidence of developing both diabetes and hypertension was 1.91-fold higher in the AUD cohort compared with the comparison cohort (10.28 vs. 5.38 per 1,000 person-years), with an aHR of 2.02 (95% CI: 1.94–2.10). The incidence rates of all three conditions were higher in males than in females in both cohorts; however, the HRs in the AUD cohort relative to the comparison cohort were higher for females than for males.

**Table 2:** Incidence of diabetes, hypertension, and diabetes and hypertension, and alcohol use disorder (AUD) cohort to compare cohort hazard ratio by gender

Disorder	AUD			Comparison			Hazard ratio (95% confidence interval)	
	Events, N	Person-years	Rate	Event no.	Person-years	Rate	Crude	Adjusted
Diabetes								
Women	1316	105,438	12.48	662	118,069	5.61	2.24 (2.04–2.45)***	2.48 (2.26–2.72)***
Men	10,377	474,137	21.89	5862	579,043	10.12	2.15 (2.08–2.22)***	2.15 (2.09–2.22)***
Overall	11,693	579,575	20.18	6524	697,111	9.36	2.14 (2.08–2.21)***	2.18 (2.11–2.24)***
Hypertension								
Women	2333	95,650	24.39	1440	111,810	12.88	1.87 (1.75–2.00)***	2.12 (1.98–2.26)***
Men	15,962	425,433	37.52	11,893	533,850	22.28	1.66 (1.62–1.70)***	1.68 (1.64–1.72)***
Overall	18,295	521,083	35.11	13,333	645,659	20.65	1.68 (1.64–1.71)***	1.72 (1.68–1.76)***
Both								
Women	730	109,548	6.66	378	120,200	3.14	2.16 (1.91–2.45)***	2.46 (2.17–2.79)***
Men	5605	506,554	11.06	3477	596,147	5.83	1.96 (1.88–2.05)***	1.98 (1.90–2.07)***
Overall	6335	616,102	10.28	3855	716,346	5.38	1.97 (1.89–2.05)***	2.02 (1.94–2.10)***

**Notes**: ***p < 0.001; Rate, per 1000 person-years

Table 3 presents the joint outcomes associated with AUD and comorbidities. Relative to the comparison cohort without comorbidity, AUD patients with CCI > 1 had elevated incidence rates of diabetes (33.14/1,000 person-years; aHR = 3.23 [95% CI: 3.08–3.40]), hypertension (42.37/1,000 person-years; aHR = 1.80 [95% CI: 1.73–1.88]), and both diabetes and hypertension (14.08/1,000 person-years; aHR = 2.54 [95% CI: 2.37–2.72]).

**Table 3:** Incidence and adjusted hazard ratio of diabetes, hypertension, and both disorders by study cohort and Charlson comorbidity index score

Disorder	Charlson comorbidity score						
	0			≥ 1			
	Events, N	Rate	HR (95% CI)	Events, n	Rate	HR (95% CI)	Interactions, p
Diabetes							< 0.0001
Comparison cohort	5385	8.81	Ref.	1139	13.26	1.18 (1.11–1.26)***	
Alcohol use disorder	9290	18.32	2.07 (2.00–2.14)***	2403	33.14	3.23 (3.08–3.40)***	
Hypertension							0.0001
Comparison cohort	10,941	19.23	Ref.	2392	31.17	1.15 (1.10–1.21)***	
Alcohol use disorder	15,446	34.03	1.75 (1.71–1.80)***	2849	42.37	1.80 (1.73–1.88)***	
Both							< 0.0001
Comparison cohort	3238	5.16	Ref.	617	6.91	1.04 (0.95–1.13)	
Alcohol use disorder	5208	9.72	1.94 (1.86–2.03)***	1127	14.08	2.54 (2.37–2.72)***	

**Notes**: Rate, per 1000 person-years; HR, hazard ratio; CI, confidence interval

CI, confidence interval; HPA, health promotion administration; HR, hazard ratio; LHID, Longitudinal Health Insurance Database; WHO, World Health Organization

## Discussion

Alcohol consumption is well known to have dose–response relationships for regulating blood pressure and glucose metabolism, contributing to the risks of developing hypertension, diabetes and hypertriglyceridemia, and may lead to cardiomyopathy, atrial fibrillation, stroke, etc. [13–20, 24]. Individuals with excessive consumption of alcohol cannot control their alcohol use, and severe-level AUD may increase the risk of vascular injury, raised blood pressure, and—when coupled with impaired β-cell function in the pancreas leading to mitochondrial dysfunction—insulin resistance and a reduction of insulin content and secretion, though the mechanism remains unknown [25–28]. Knott et al. reported in a meta-analysis that alcohol consumption may interact with dyslipidemia to increase the risk of diabetes [7]. High alcohol consumption causes insulin resistance, raises diabetes risk, and worsens other symptoms of diabetes [12]. Excessive alcohol consumption leads to inflammation and oxidative injury of the endothelium, and endothelium-dependent nitric oxide production is inhibited [29]. Heavy alcohol use is thus associated with the development of primary hypertension [30].

Most studies have investigated the association between alcohol consumption and risk of hypertension and diabetes. Our study investigated both disorders and found nearly 1.6-fold more hypertension cases than diabetes cases (18,295 vs. 11,693 cases, or 35.11 vs. 20.18 per 1000 person-years). We also found that 6,335 cases (10.28 per 1000 person-years) had developed both hypertension and diabetes in the AUD cohort. In contrast, the corresponding outcomes in the comparison cohort were lower. Our findings demonstrated that risk of developing both disorders was higher in females than in males and increased with comorbidities.

A systematic review based on 12 cohort studies from the U.S., Japan, and Korea found that the risk of hypertension for those who had used 100 g of alcohol daily was slightly higher for female than for male alcohol users (RR, 2.81 vs. 2.47) [31]. Another review based on 16 prospective studies found that heavy alcohol consumption of 31–40 g/day had an RR of 1.19 for developing hypertension [32]. Limited research has examined the hypertension risk or diabetes risk, particularly among individuals with AUD. A Korean study that followed up with community participants 40–69 years old found a nearly 2-fold higher risk of hypertension in heavy drinkers [33]. A cross-sectional survey in Xu Zhou, China, interviewed 36,157 adults using the Michigan AUD Screening Test and found an alcohol dependence rate of 11.56% [34]. Participants with severe AUD were at a higher risk of developing hypertension, with an odds ratio of 1.83 (95% CI = 1.40–2.41) compared with those with moderate AUD. In the XuZhou data, the prevalence of alcohol dependence was nearly 13-fold higher in males than in females (22.02% vs. 1.74%). Herein, there were 5.4-fold more male than female patients with AUD. The incidence of hypertension was higher in males than in females in both the cohorts. The ratio of the AUD cohort to the comparison cohort HR was higher for females than males, indicating that females who consumed excess alcohol were more likely to be at a higher risk of developing hypertension than males.

Similarly, the HRs for developing diabetes, or both hypertension and diabetes among patients with AUD were higher for females than for males herein. A Swedish population study reported that high consumption of alcohol was also associated with a slightly higher diabetes risk for females than for males (OR, 2.41 vs. 2.03) [35]. The Mendelian randomization study in China found that an increase of one unit (22.05 g ethanol) of consumption is associated with an enhanced diabetic relative risk of 1.32 (*p* = 0.014) in males and 1.40 (*p* = 0.377) in females [9]. The gender difference in the risks of hypertension and diabetes may be associated with the impact of alcohol on the sex hormones of a patient, leading to the discrepancy in disease risk between genders [36]. Moreover, it is important to determine whether the risks of developing diabetes and hypertension are additionally increased among patients with AUD who have other comorbidities measured by the CCI. Herein, AUD patients with a higher CCI were at increased risk of hypertension, diabetes, or both hypertension and diabetes. Comorbidities are likely to play a role in the development of hypertension and diabetes in patients with AUD. The absolute risk is higher for developing hypertension than for developing diabetes, but the relative risk is higher for developing diabetes.

### Strengths and limitations

This study had the advantage of using a sizable, longitudinal population database of good quality to conduct a 16-year follow-up study that evaluated the relationships between AUD and the risk of diabetes and hypertension. With a long follow-up period, the large sample size guaranteed more unbiased, reliable estimations, which were superior to those of previous studies [35, 37, 38]. The statistical technique of propensity score matching for establishing study cohorts reduced the selection bias due to confounding factors. Moreover, gender differences were considered when examining whether an interaction between gender and alcohol existed.

This study has some limitations. First, information on some potential confounding factors was unavailable in the claims data, such as education, work pressure, body weight, and lifestyles [6]. Among these factors, lifestyle information was likely more important than others. Patients with AUD generally smoke tobacco and their diets are nutritionally deficient. Smoking is also a risk factor for complications of hypertension and diabetes [38–41]. In general, females in Taiwan rarely smoke [42]. However, females with alcohol dependence were more likely to be tobacco smokers than females in the comparison cohort. We were unable to evaluate the contribution of smoking to the risk of hypertension and diabetes, in addition to alcohol, particularly in females with AUD. Second, the study population was derived from the people of Taiwan only; therefore, generalizing our findings to other populations requires further research. Risks may vary among races and populations [9, 43].

## Conclusions

This study showed that there were nearly 5.4-fold more males than females diagnosed with AUD, mainly at younger ages. Gender differences were observed in the risk of developing both hypertension and diabetes in patients with AUD. Our data also showed that caution is needed for alcoholic patients with comorbidities, which may further increase health risks.

## Data Availability

The datasets used in this study are available from the Ministry of Health and Welfare, Taiwan, on reasonable request.

## Abbreviations

aHR: Adjusted HR
AUD: Alcohol use disorder
CCI: Charlson Comorbidity Index
CI: Confidence intervals
diabetes: Diabetes
HR: Hazard ratios
ICD-9-CM: International Classification of Diseases, Ninth Revision, Clinical Modification
ICD-10-CM: International Classification of Diseases, Tenth Revision, Clinical Modification
LHID: Longitudinal Health Insurance Database
RR: Relative risks
